# Electron hydrodynamics in anisotropic materials

**DOI:** 10.1038/s41467-020-18553-y

**Published:** 2020-09-18

**Authors:** Georgios Varnavides, Adam S. Jermyn, Polina Anikeeva, Claudia Felser, Prineha Narang

**Affiliations:** 1 Harvard John A. Paulson School of Engineering and Applied Sciences, Harvard University, Cambridge, MA 02138 USA; 2 Department of Materials Science and Engineering, Massachusetts Institute of Technology, Cambridge, MA 02139 USA; 3 Research Laboratory of Electronics, Massachusetts Institute of Technology, Cambridge, MA 02139 USA; 4 Center for Computational Astrophysics, Flatiron Institute, New York, NY 10010 USA; 5 Max-Planck-Institut für Chemische Physik fester Stoffe, Dresden, 01187 Germany

**Keywords:** Condensed-matter physics, Fluid dynamics

## Abstract

Rotational invariance strongly constrains the viscosity tensor of classical fluids. When this symmetry is broken in anisotropic materials a wide array of novel phenomena become possible. We explore electron fluid behaviors arising from the most general viscosity tensors in two and three dimensions, constrained only thermodynamics and crystal symmetries. We find nontrivial behaviors in both two- and three-dimensional materials, including imprints of the crystal symmetry on the large-scale flow pattern. Breaking time-reversal symmetry introduces a non-dissipative Hall component to the viscosity tensor, and while this vanishes for 3D isotropic systems we show it need not for anisotropic materials. Further, for such systems we find that the electronic fluid stress can couple to the vorticity without breaking time-reversal symmetry. Our work demonstrates the anomalous landscape for electron hydrodynamics in systems beyond graphene, and presents experimental geometries to quantify the effects of electronic viscosity.

## Abstract (web summary)

In some materials electrons can behave hydrodynamically, exhibiting phenomena associated with classical viscous fluids. In this theory work, the authors show that the symmetries of the crystal lattices in which the electrons reside can lead to additional unique hydrodynamic effects.

## Introduction

Theoretical and experimental studies have revealed that electrons in condensed matter can behave hydrodynamically, exhibiting fluid phenomena such as Stokes flow and vortices^1–9^. Unlike classical fluids, preferred directions inside crystals lift isotropic restrictions, necessitating a generalized treatment of electron hydrodynamics. While anisotropic viscous flows have been studied in geophysics^10^, their prominence in condensed matter has yet to be explored. This is of particular importance, given the recent demonstration of hydrodynamic behavior in three-dimensional materials such as Weyl semimetals^11,12^. Electron hydrodynamics is observed when microscopic scattering processes conserve momentum over time- and length scales that are large compared to those of the experimental probe. However, even as momentum is conserved, free energy may be dissipated from the electronic system, giving rise to a measurable viscosity in the electron flow^12–18^.

When momentum is conserved, a fluid obeys Cauchy’s laws of motion^19^

1 $$\rho \dot{{u}_{i}}={\partial }_{j}{\tau }_{ji}+\rho {f}_{i}$$

2 $$\rho \dot{{\sigma }_{i}}={\partial }_{j}{m}_{ji}+\rho {l}_{i}+{\epsilon }_{ijk}{\tau }_{jk},$$

where *u* and *ρ* are the fluid velocity and density, *f* and *l* are body forces and couples, *τ* and *m* are the fluid stress and couple stress, and *σ* is the intrinsic angular momentum density (internal spin). The superscript dot denotes the material derivative, $\dot{x}={\partial }_{t}x \, +{u}_{j}{\partial }_{j}x$, and *ϵ* is the rank-3 alternating tensor. We assume couple stresses and body couples to be zero, but allow for body forces of the form *ρf*_*i*_ = −*R*_*ij*_*u*_*j*_, where *R* is a rank-2, positive–semidefinite tensor that is inversely proportional to a microscopic momentum-relaxing lifetime. In steady state and at experimentally accessible Reynolds numbers^17,20^, this implies that the stress tensor is symmetric^19^. In this limit, electron fluids obey the modified Navier–Stokes equation

3 $$\rho {u}_{j}{\partial }_{j}{u}_{i}=-{\partial }_{i}p+{\partial }_{j}{\tau }_{ji}-{R}_{ij}{u}_{j},$$

where *τ* is symmetric. Note that in electron fluids, current density is analogous to the fluid velocity, and voltage drops are analogous to changes in pressure. Assuming that the fluid velocity is much smaller than the electronic speed of sound, *u* ≪ *c*_s_, the electron fluids are nearly incompressible, thus

4 $${\partial }_{i}{u}_{i}=0.$$

In this limit, *ρ* is a constant, which we take to be unity. Since the fluid stress appears in a divergence, it is defined only up to a constant, which we choose to make *τ* vanish when *u* is uniformly zero^21,22^. We further assume that the fluid stress vanishes for uniform flow, so that it is only a function of the velocity gradient.

Without further loss of generality, the constitutive relation is written to the first order as^21^

5 $${\tau }_{ij}={A}_{ijkl}{\partial }_{l}{u}_{k},$$

where *A* is the fluid viscosity, a rank-4 tensor relating the fluid velocity gradient (∂_*j*_*u*_*i*_) and the fluid stress. Since we take *τ* to be symmetric, *A* is invariant under permutation of its first two indices, i.e., *A*_*ijkl*_ = *A*_*jikl*_^21,22^. Viscosity is represented as the sum of three rank-4 tensor basis elements^23^, summarized in Table 1

6 $${A}_{(ij)kl}={\alpha }_{((ij)(kl))}+{\beta }_{[(ij)(kl)]}+{\gamma }_{(ij)[kl]}.$$

**Table 1:** Rank-4 tensors used as orthogonal basis elements for the viscosity tensor.

Tensor	Tensor symmetries				Indep. comp.	
	*i* ↔ *j*	*k* ↔ *l*	*ij* ↔ *kl*	Type	3D	2D
*α*_((*ij*)(*kl*))_	+	+	+	Proper	21	6
*β*_[(*ij*)(*kl*)]_	+	+	−	Pseudo	15	3
*γ*_(*ij*)[*kl*]_	+	−	N/A	Pseudo	18	3

Even and odd symmetries are represented using parentheses and square brackets, respectively. The fifth column specifies whether the tensor changes sign under mirror operations.

Tensor *α* describes dissipative behavior respecting both stress symmetry and objectivity, i.e., *α*_*ijkl*_ = *α*_*jikl*_ = *α*_*klij*_. Tensor ***β*** on the other hand, describes nondissipative Hall viscosity^7,23–27^, i.e., *β*_*ijkl*_ = −*β*_*klij*_, and is nonzero only when time-reversal symmetry is broken. Finally, ***γ*** breaks stress objectivity, i.e., *γ*_*ijkl*_ = −*γ*_*ijlk*_, coupling fluid stress to the vorticity. The fifth column in Table 1 specifies whether the tensor is defined according to a handedness convention.

In classical fluids, the added consideration of rotational invariance requires *A* to be isotropic, reducing it to the form

7 $${A}_{ijkl}= 	\, \lambda {\delta }_{ij}{\delta }_{kl}+\mu \left({\delta }_{il}{\delta }_{jk}+{\delta }_{jl}{\delta }_{ik}\right)\\ \, 	+{{\mathcal{B}}}_{1}\left({\epsilon }_{ik}{\delta }_{jl}+{\delta }_{ik}{\epsilon }_{jl}\right)+{\Gamma }_{1}{\delta }_{ij}{\epsilon }_{kl},$$

where *δ* is the Kronecker delta, *ϵ* is the rank-2 alternating tensor, and the Lamé parameters *λ* and *μ* can be identified as the two independent components of the proper tensor *α*. In the incompressible case, *λ* does not contribute to the stress^21^. ${{\mathcal{B}}}_{1}$ and Γ_1_ are constants parameterizing terms with the symmetry of *β* and *γ*, respectively. Since *β* and *γ* are pseudotensors, the last three terms in Eq. (7) are only nonzero in two dimensions^23,24^.

In crystals, however, there exist preferred directions and we cannot assume rotational invariance. Instead, we must consider the effect of the crystal symmetry given by Neumann’s principle^28,29^, which requires that physical properties described by rank-4 tensors, such as viscosity, remain invariant under the transformation law

8 $${A}_{ijkl}^{\prime}=| s{| }^{\eta }{s}_{im}{s}_{jn}{s}_{ko}{s}_{lp}{A}_{mnop},$$

where *s* is the space representation of any given point group symmetry of the crystal, ∣*s*∣ = ±1 is the determinant of the symmetry operation, and *η* = 0 for proper tensors and *η* = 1 for pseudotensors.

Although Eq. (8) relates different components of the viscosity tensor, further constrains must be imposed to ensure that the viscosity tensor never does positive work in Eq. (3), so that for any velocity field *u* in *d* dimensions

9 $$\int {u}_{i}{\partial }_{j}({A}_{ijkl}{\partial }_{k}{u}_{l}){d}^{d}{\bf{r}}\le 0.$$

Letting the Fourier transform of *u* be

10 $$\tilde{{\bf{u}}}({\bf{q}})=\int {e}^{i{\bf{q}}\cdot {\bf{r}}}u({\bf{r}}){d}^{d}{\bf{r}}$$

in *d* dimensions, we find

11 $$\int {q}_{j}{q}_{k}{\tilde{u}}_{i}^{* }({\bf{q}}){\tilde{u}}_{l}({\bf{q}}){A}_{ijkl}{d}^{d}{\bf{q}}\ge 0.$$

This is satisfied when *A*_*ijkl*_ has a positive definite biquadratic form in *il* and *jk*, so we impose this constraint in addition to *ij* symmetry and crystal symmetry.

Viscosity tensors are then randomly generated to satisfy the aforementioned constraints, allowing for normalized numerical deviations from isotropy lower than order unity. The viscosity tensor is assumed to be spatially uniform in all cases. To demonstrate the differences between these general viscosity tensors and those more strongly constrained by symmetry, we solve for the velocity and pressure of low Reynolds number flows in several geometries. The parameterization of the viscosity tensor in Eq. (6) allows us to explore the effects of breaking stress objectivity and time-reversal symmetry. We highlight the effects of symmetry in the last two indices (*kl*) because it implies that the stress only couples to the strain rate (∂_*k*_*u*_*l*_ + ∂_*l*_*u*_*k*_) and not to the vorticity (∂_*k*_*u*_*l*_ − ∂_*l*_*u*_*k*_). This is a property of classical fluids, which means that rigid–rotational flows are stress-free, and hence are only sensitive to rotation via weaker effects like the Coriolis force. Below, we demonstrate that with more general viscosity tensors, this is not the case, and that the resulting rotational stresses can be probed in experimentally accessible geometries.

## Results

### Effect of anisotropy

**Fig. 1:**
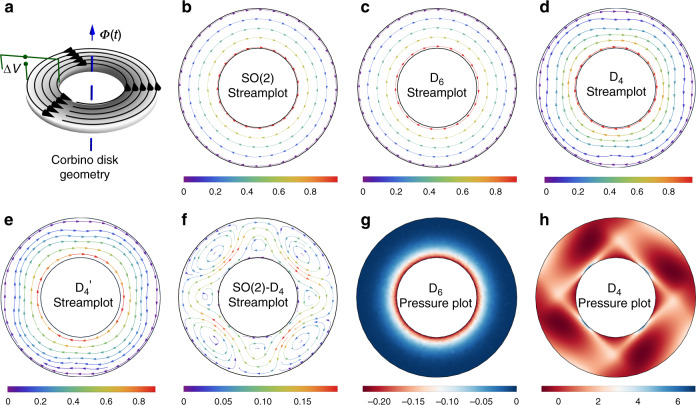
Effect of viscosity tensor anisotropy on rotational flow in an annulus. **a** Corbino disk geometry schematic. The time-varying magnetic flux, *Φ*(*t*), acting on a voltage drop, Δ*V*, gives rise to a Lorentz force, inducing rotational electron flow. **b** Steady-state streamplot plot using an isotropic (SO(2)) viscosity tensor. Streamplots using **c** hexagonal (*D*_6_), and **d** square (*D*_4_) viscosity tensors. **e** Streamplot using square (*D*_4_) viscosity tensor, allowing for momentum-relaxing body force terms equal to ∣*R*∣*L*^2^/∣*A*∣ = 0.1. **f** Difference in steady-state streamplot between isotropic and *D*_4_ viscosity tensors, highlighting the emergence of steady-state vortices. Steady-state pressure plot using **g** *D*_6_ and **h** *D*_4_ viscosity tensors, illustrating the breaking of azimuthal symmetry in the latter. Color scales indicate the magnitude of the velocity vector field (**b**–**f**) and pressure field (**g**, **h**).

We first consider rotational flow in an annulus with inner radius *R*_inner_ = 1 and outer radius *R*_outer_ = 2 (Fig. 1). We apply a no-slip condition to the outer boundary, allow the inner boundary to rotate with unit angular velocity *ω* = 1, and solve for the steady-state flow at Reynolds number

12 $${\rm{Re}}\equiv \frac{\omega {R}_{{\rm{inner}}}^{2}}{\left|A\right|}=0.3,$$

where

13 $${\left|A\right|}^{2}={A}_{ijkl}{A}_{ijkl}.$$

The zero-pressure point is fixed at the bottom of the annulus. Experimentally, such rotational flows can be achieved by threading a time-varying magnetic flux through a Corbino disk geometry^30,31^ (Fig. 1a). For a fluid with an isotropic viscosity, the steady-state velocity field rotates rigidly with the angular velocity set by the inner boundary condition (Fig. 1b).

**Fig. 2:**
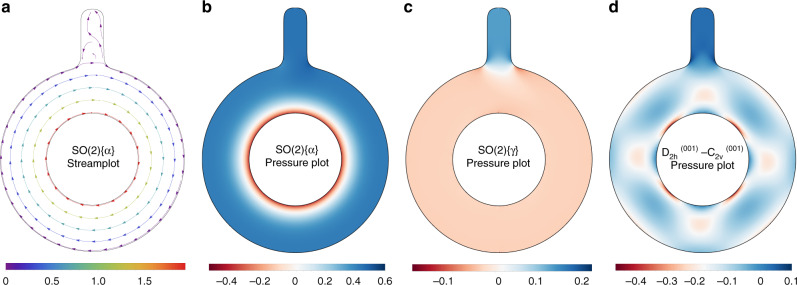
Proposed setup to quantify the effect of viscosity tensor asymmetry and Hall coefficient. Steady-state **a** streamplot and **b** pressure plot using viscosity tensor SO(2){*α*}. **c** Difference in steady-state pressure between viscosity tensor SO(2){*α*} and the same with additional stress objectivity-breaking terms (SO(2){*γ*}). The asymmetry introduces an additional pressure-like contribution, which can be directly measured. **d** Difference in steady-state pressure between the *D*_2*h*_ and *C*_2*v*_ viscosity tensors along the *ab* plane, showing a similar pressure drop in the gauge, despite the anisotropic behavior inside the annulus. Color scales indicate the magnitude of the velocity vector field (**a**) and pressure field (**b**–**d**).

To investigate the effects of anisotropy in two-dimensional materials, we consider materials with *D*_6_ (hexagonal) and *D*_4_ (square) symmetry. Notably, *D*_6_ materials do not deviate from isotropic behavior (Fig. 1(c)), consistent with experimental observations for graphene^9,17^. We note that 2D materials with *C*_3_ (threefold), *C*_6_ (sixfold), and *D*_3_ (triangular) symmetry also exhibit isotropic viscosity tensors (see Supplementary Methods). By contrast, the flow deviates considerably from isotropic behavior in *D*_4_ materials (Fig. 1d). We repeat the calculation, allowing for a momentum-relaxing body force equal to ∣*R*∣*L*^2^/∣*A*∣ = 0.1, illustrating that the deviation from isotropy remains observable (Fig. 1e). We assume ***R*** → 0 for the rest of the paper, and investigate its effects and symmetry in Supplemenentary Figs. 1 and 2. Figure 1f shows the steady-state velocity flow difference between the isotropic case and *D*_4_ materials. We observe steady-state vortices emerging at  ~15% of the bulk flow rate overlaid onto the isotropic velocity field. While the steady-state pressure field in *D*_6_ materials mirrors that of an isotropic fluid (Fig. 1g), the pressure field in *D*_4_ materials also exhibits four vortices (Fig. 1h), with orientation set by the underlying crystal axes.

### Effect of asymmetry

We next examine the importance of symmetry in the last two indices of the viscosity tensor. We calculate the flow profile for the annulus in Fig. 1 scaled by a factor of two, equipped with a pressure gauge, as shown in Fig. 2a. The pressure gauge is a channel with no-slip boundary conditions, allowing us to measure the difference between the flow and a nearly stationary fluid. To isolate the effects of ${{\mathcal{B}}}_{1}$ and Γ_1_ in Eq. (7), Fig. 2a, b shows the flow and pressure fields in the annulus for a material with isotropic viscosity tensor where both ${{\mathcal{B}}}_{1}$ and Γ_1_ have been set to zero (SO(2){*α*}). These are nearly unchanged inside the annulus as compared to Fig. 1b, g, with a constant pressure in the gauge. Allowing for nonzero stress-breaking components, i.e., using a material with isotropic viscosity for ${{\mathcal{B}}}_{1}=0$ and Γ_1_ = 0.25 (SO(2){*γ*}), we observe a significant pressure buildup near the gauge. This is due to the shear stress between the rotating and stationary fluids, while the pressure within the gauge itself is nearly uniform, as shown in Fig. 2c.

To quantify the pressure difference between SO(2){*α*} and SO(2){*γ*}, note that the pressure is fixed to zero at a point *p*, the bottom of the annulus domain. The pressure in the gauge may be written as the path integral

14 $${p}_{{\rm{gauge}}}=\int_{p}^{g}\nabla p\cdot d{\bf{s}},$$

where *g* is a point in the gauge. At low Reynolds numbers, we may neglect *u*_*j*_∂_*j*_*u*_*i*_ in Eq. (3), to find in the steady state

15 $$\nabla p={A}_{ijkl}{\partial }_{i}{\partial }_{k}{u}_{l}.$$

16 $${p}_{{\rm{gauge}}}=\int_{p}^{g}{A}_{ijkl}{\partial }_{i}{\partial }_{k}{u}_{l}d{s}_{j}.$$

Taking into account Eq. (7) and noting that the changes in fluid flow are negligible, we find

17 $$\Delta {p}_{{\rm{gauge}}}=\int_{p}^{g}\Delta {A}_{ijkl}{\partial }_{i}{\partial }_{k}{u}_{l}d{s}_{j}={\Gamma }_{1}\int_{p}^{g}{\partial }_{i}\omega d{s}_{i}={\Gamma }_{1}\Delta \omega ,$$

where *ω*_*i*_ = *ϵ*_*ijk*_∂_*j*_*u*_*k*_ is the vorticity of the flow. For the geometry used, we find Δ*p*_gauge_ = 0.15, vorticity in the gauge is zero, and that in the annulus is 0.6, so Δ*ω* = 0.6 (Fig. 2c). Since we chose Γ_1_ = 0.25, we see that in this setup, the pressure gauge (Δ*p*_gauge_ = Γ_1_Δ*ω* = 0.25 × 0.6 = 0.15) is directly sensitive to the asymmetry in the viscosity, which couples the rotation directly to the pressure field and the stress. We note that the same setup is sensitive to Hall viscosity coefficients for time-reversal broken systems, i.e., for the case where both ${{\mathcal{B}}}_{1}$ and Γ_1_ are nonzero, the pressure gauge generalizes to

18 $$\Delta {p}_{{\rm{gauge}}}=\int_{p}^{g}\Delta {A}_{ijkl}{\partial }_{i}{\partial }_{k}{u}_{l}d{s}_{j}=({{\mathcal{B}}}_{1}+{\Gamma }_{1})\Delta \omega .$$

While time-reversal and stress objectivity-breaking terms persist in two-dimensional isotropic materials, the handedness of the pseudotensor implies that mirror operations set them to zero in 3D. This can be directly observed by comparing low- and high-symmetry three-dimensional crystals. We consider the same rotational flow along the *ab* crystal plane of orthorhombic materials, such as the hydrodynamically reported Weyl semimetal WP2^11,12^. Along this plane, the difference between the two viscosity tensors can be parameterized as follows:

19 $$\begin{array}{l}{A}_{ijkl}^{{C}_{2v}^{(001)}}={A}_{ijkl}^{{D}_{2h}^{(001)}}+{\Gamma }_{2}{\delta }_{ij}{\epsilon }_{kl}+{\Gamma }_{3}{\sigma }_{ij}^{z}{\epsilon }_{kl}\\ \hspace{1em}+{{\mathcal{B}}}_{2}\left({\delta }_{li}{\epsilon }_{jk}-{\epsilon }_{li}{\delta }_{jk}\right)+{{\mathcal{B}}}_{3}\left({\delta }_{ij}{\sigma }_{kl}^{x}-{\sigma }_{ij}^{x}{\delta }_{kl}\right),\end{array}$$

where ${{\mathcal{B}}}_{2}$, ${{\mathcal{B}}}_{3}$, Γ_2_, and Γ_3_ are constants parameterizing terms with the symmetry of *β* and *γ*, respectively, *σ*^*x*^ and *σ*^*z*^ are Pauli matrices. Figure 2d shows the pressure difference between a material with *D*_2*h*_ symmetry and one with *C*_2*v*_ symmetry (for ${{\mathcal{B}}}_{2}={{\mathcal{B}}}_{3}={\Gamma }_{3}=0$ and Γ_2_ = 0.25), indicating the same pressure buildup as in Fig. 2c inside the gauge along with a nontrivial pressure structure in the annulus.

### 2D flows in 3D crystals

**Fig. 3:**
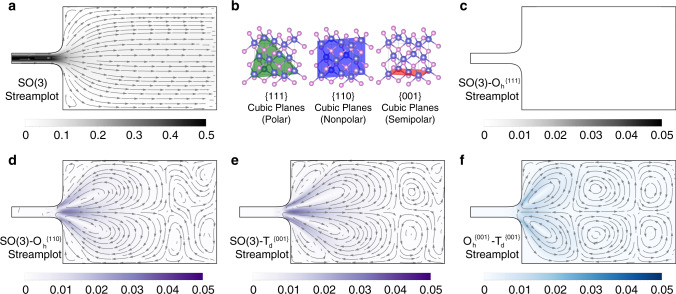
Three-dimensional projected flows through an expanding geometry. **a** Steady-state streamplot using an isotropic (SO(3)) viscosity tensor. **b** High-symmetry family of planes in cubic crystals. In crystals with *T*_d_ (tetrahedral) symmetry, these are further identified as polar {111}, nonpolar {110}, and semipolar {001}. Difference in the steady-state streamplot between using an isotropic viscosity tensor and using **c** (111)-projected, **d** (110)-projected, and **e** (001)-projected viscosity tensors of cubic crystals with *T*_d_ symmetry. Note that **c** shows no difference. **f** Effect of viscosity tensor asymmetry difference between *O*_h_ and *T*_d_ crystals along a semipolar {001} plane. Color scales indicate the magnitude of the velocity vector field.

Finally, we consider flow through an expanding channel along high-symmetry planes in 3D. This geometry has been proposed as a diagnostic of electron hydrodynamics because it naturally generates vortices, not present in ordinary ohmic flow. The case with isotropic viscosity is shown in Fig. 3a, where the small vortices that form in the corners are clearly detached from the bulk of the flow. We consider the *T*_d_ (tetrahedral) and *O*_h_ (cubic) point groups. In particular, we consider flows along the polar {111}, nonpolar {110}, and semipolar {001} family of planes (Fig. 3b)

20a $${A}_{ijkl}^{{T}_{{\rm{d}}}^{(111)}}={A}_{ijkl}^{{O}_{{\rm{h}}}^{(111)}}+{{\mathcal{B}}}_{4}\left({\sigma }_{ij}^{x}{\sigma }_{kl}^{z}-{\sigma }_{ij}^{z}{\sigma }_{kl}^{x}\right)+{\Gamma }_{4}{\delta }_{ij}{\epsilon }_{kl}$$

20b $${A}_{ijkl}^{{T}_{{\rm{d}}}^{(110)}}={A}_{ijkl}^{{O}_{{\rm{h}}}^{(110)}}$$

20c $${A}_{ijkl}^{{T}_{{\rm{d}}}^{(001)}}={A}_{ijkl}^{{O}_{{\rm{h}}}^{(001)}}+{{\mathcal{B}}}_{5}\left({\sigma }_{ij}^{z}{\delta }_{kl}^{z}-{\delta }_{ij}{\sigma }_{kl}^{z}\right)+{\Gamma }_{5}{\sigma }_{ij}^{x}{\epsilon }_{kl}.$$

Along these planes, the difference between the two viscosity tensors can be parameterized according to Eqs. (20a), (20b), (20c). We impose fully developed (parabolic) inlet and outlet flows with constant discharge, and solve for the steady-state flow at low Reynolds number. Figure 3c shows the difference between the flow in an isotropic material and the flow in a cubic material along a {111} close-packed plane, which exhibits rotational invariance. Along the nonpolar {110} planes, terms with *β* and *γ* symmetry vanish. However, ${{A}}^{{{O}}_{{\rm{h}}}^{(110)}}$ is anisotropic along this plane, with Fig. 3d showing the difference in flow between the isotropic case. Finally, along the semipolar {001} family of planes, the viscosity tensor is both anisotropic (Fig. 3e) and asymmetric. Figure 3f quantifies the additional vortices generated by the asymmetry at  ~10%, for ${{\mathcal{B}}}_{5}=0$ and Γ_5_ = 0.25.

## Discussion

We found that electron fluids in crystals with anisotropic and asymmetric viscosity tensors can exhibit steady-state fluid behaviors not observed in classical fluids. In 3D, discrete deviations from isotropy allow the fluid stress to couple to the fluid vorticity with or without breaking time-reversal symmetry, for the case of Hall viscosity and objectivity-breaking viscosity, respectively. Recent measurements of spatially resolved flows^9,17,32^ suggest that these effects can be directly observed in systems beyond graphene. Our findings further hint at potential applications. For instance, the pressure gauge in Fig. 2 could be used as a magnetometer, converting a time-varying magnetic flux through a modified Corbino disk geometry into current in the annulus, and ultimately into a voltage drop between it and the gauge. Our work highlights the importance of crystal symmetry on electronic flow, and invites further exploration of time-dependent flows in systems with internal spin degrees of freedom and asymmetric stress tensors.

## Supplementary information

Supplementary Information

Peer Review File

## Data Availability

The authors declare that the main data supporting the findings of this study are available within the article and its Supplementary Information files.

## Code availability

Code available upon request from the authors.
