# Supplementary material for: Electron hydrodynamics in anisotropic materials
Source: Nat Commun. 2020 Sep 18;11:4710. doi: 10.1038/s41467-020-18553-y (PMC7501241; doi:10.1038/s41467-020-18553-y)
Supplement: Supplementary file 1 — Supplementary Information [file 41467_2020_18553_MOESM1_ESM.pdf]

Supplementary Information for Electron Hydrodynamics in Anisotropic Materials

Georgios Varnavides,[1,2,3,*] Adam S. Jermyn,[4,*] Polina Anikeeva,[2,3] Claudia Felser,[5] and Prineha Narang[1,†]
[1]*John A. Paulson School of Engineering and Applied Sciences, Harvard University, Cambridge, MA,USA*
[2]*Department of Materials Science and Engineering, Massachusetts Institute of Technology, Cambridge, MA, USA*
[3]*Research Laboratory of Electronics, Massachusetts Institute of Technology, Cambridge, MA, USA*
[4]*Center for Computational Astrophysics, Flatiron Institute, New York, NY 10010, USA*
[5]*Max-Planck-Institut für Chemische Physik fester Stoffe, Dresden, Germany*

Supplementary Methods

Stress Tensor Symmetries. The viscosity tensors used in the manuscript are all invariant under permutation of the first two indices, i.e. $A_{ijkl} = A_{jikl}$. This is a direct consequence of the stress tensor being symmetric under the assumptions and conditions used, which we elaborate on in this section. The anti-symmetric part of the stress tensor, is constrained by Cauchy's laws of motion [1]

$$\rho \dot{u}_i = \partial_j \tau_{ji} + \rho f_i \tag{1}$$
$$\rho \dot{\sigma}_i = \partial_j m_{ji} + \rho l_i + \epsilon_{ijk}\tau_{jk}, \tag{2}$$

where the symbols have the same meaning as in the manuscript.

In the absence of internal spin degrees of freedom $\boldsymbol{\sigma}$, body couples $\mathbf{l}$, and stress couples $\mathbf{m}$, eq. (2) necessitates that the anti-symmetric part of the stress tensor, given by the pseudo-vector $T_i^{\mathrm{A}} = \epsilon_{ijk}\tau_{jk} = 0$, must vanish.

If we allow for nonzero internal spin, then eq. (2) instead specifies:

$$T_i^{\mathrm{A}} = \epsilon_{ijk}\tau_{jk} = \rho\dot{\sigma}_i. \tag{3}$$

Recall that the superscript dot denotes the material derivative, $\dot{\sigma}_i = \partial_t \sigma_i + u_j \partial_j \sigma_i$, which for low Reynolds number flows can be approximated as simply the partial time derivative, $\dot{\sigma}_i \approx \partial_t \sigma_i$, which by definition must vanish at steady-state. Therefore, at the experimentally accessible conditions we consider in the paper, the anti-symmetric part of the stress tensor again must vanish at steady-state.

In the most general case where we consider stress couples (and/or body couples), the anti-symmetric part of the stress tensor does not vanish. To account for it, we need to augment our constitutive relations to read [1, 2]:

$$\tau_{ij} = A_{ijkl}\partial_l u_k + \xi_{[ij]k}\sigma_k \tag{4}$$
$$m_{ij} = B_{ijkl}\partial_l u_k + \kappa_{[ij]k}\sigma_k, \tag{5}$$

where $\mathbf{B}$ is a rank-4 tensor, and $\boldsymbol{\xi}$ and $\boldsymbol{\kappa}$ are rank-3 tensors anti-symmetric with respect to their first two indices.

Breaking stress symmetry, therefore allows one to express the viscosity tensor in an expanded basis set [2]:

$$A_{ijkl} = \alpha_{((ij)(kl))} + \beta_{[(ij)(kl)]} + \gamma_{(ij)[kl]} + \delta_{[ij](kl)} + \varepsilon_{([ij][kl])} + \zeta_{[[ij][kl]]}. \tag{6}$$

Finally, we note the interesting observation that the stress tensor always appears in a divergence in eq. (1). This suggests that there is an ambiguity in the choice of stress tensor, and in-fact one can always write a *physically indistinguishable* symmetrized tensor [3–5]:

$$\tilde{\tau}_{(ij)} = \tau_{ij} + \partial_k \chi_{i[jk]}, \tag{7}$$

where χ is a rank-3 tensor anti-symmetric in j and k, i.e. $\chi_{ijk} = -\chi_{ikj}$. While it's straight-forward to show the divergence of the additional term in eq. (7) vanishes, we need to establish this symmetrizes $\tilde{\boldsymbol{\tau}}$. Consider the following choice:

$$\chi_{ijk} = \partial_i \phi_{[jk]} + \partial_j \phi_{[ik]} - \partial_k \phi_{[ij]}, \tag{8}$$

where $\boldsymbol{\phi}$ is anti-symmetric, and chosen as the solution to Poisson's equation with the anti-symmetric part of the stress tensor as a source:

$$\nabla^2 \phi_{[ij]} = \frac{1}{2}\left(\tau_{ij} - \tau_{ji}\right). \tag{9}$$

Substituting eqs. (8) and (9) in eq. (7), we obtain [5]:

$$\tilde{\tau}_{(ij)} = \tau_{ij} + \partial_k\left(\partial_i \phi_{[jk]} + \partial_j \phi_{[ik]}\right) - \nabla^2 \phi_{[ij]} \tag{10}$$
$$= \frac{1}{2}\left(\tau_{ij} + \tau_{ji}\right) + \partial_k\left(\partial_i \phi_{[jk]} + \partial_j \phi_{[ik]}\right), \tag{11}$$

which is symmetric as desired.

Irreversible Thermodynamics. Viscosity is a non-equilibrium transport tensor which we analyze using irreversible thermodynamics. The entropy production for a system evolving irreversibly from a non-equilibrium state is given by [6–8]:

$$\frac{dS}{dt} = \sum_i \frac{\partial S}{\partial \xi_i}\frac{\partial \xi_i}{\partial t} = \sum_i X_i J_i, \tag{12}$$

where $X_i = \partial S/\partial \xi_i$ are generalized (intensive) forces, and $J_i = \partial \xi_i/\partial t$ are the thermodynamic fluxes of the conjugate generalized (extensive) displacements ξ_i. The thermo-

* These authors contributed equally to this work
† Electronic address: prineha@seas.harvard.edu

dynamic fluxes can be expressed as linear combinations of the generalized forces via Onsager's phenomenological equations: $\mathbf{J} = \boldsymbol{L}\mathbf{X}$, where $\mathbf{L}$ is a matrix of kinetic coefficients. The Onsager reciprocal relations postulate $\mathbf{L}$ is a symmetric matrix, concluding that entropy production is given by:

$$\frac{dS}{dt} = \sum_{ij} L_{ij} X_i X_j, \tag{13}$$

We note that Onsager derived that the kinetic coefficients matrix is symmetric, due to the microscopic reversibilty of equilibrium states, but that the symmetry is not necessitated by eq. (13) [9, 10]. Consider a general kinetic coefficients matrix given by the sum of a symmetric and antisymmetric component $\mathbf{L} = \mathbf{L}^{\mathrm{S}} + \mathbf{L}^{\mathrm{A}}$. The antisymmetric component does not contripute to dissipation, i.e. it describes isentropic processes[10]:

$$\frac{dS}{dt} = \sum_{ij} L^{\mathrm{A}}_{ij} X_i X_j = 0, \tag{14}$$

We identify the generalized force as the fluid stress $\boldsymbol{\tau}$ and the generalized displacement as the velocity gradient $\partial_j u_i$. We split the viscosity tensor as in the manuscript in the three tensors: $A_{(ij)kl} = \alpha_{((ij)(kl))} + \beta_{[(ij)(kl)]} + \gamma_{(ij)[kl]}$. Equation (13) then imposes additional 'major' symmetries on $\boldsymbol{\alpha}$, and $\boldsymbol{\beta}$, i.e. under permutation of $ij \leftrightarrow kl$, $\alpha_{ijkl} = \alpha_{klij}$ and $\beta_{ijkl} = -\beta_{klij}$. The 'odd' Hall viscosity tensor, identified as $\boldsymbol{\beta}$, which breaks time-reversal symmetry, is dissipationless. Tensor $\boldsymbol{\gamma}$ which breaks stress objectivity is not symmetric under the permutation of $ij \leftrightarrow kl$, but rather spans a two-dimensional subspace with both 'even' and 'odd' components [2].

Onsager's regression hypothesis can be put on firmer footing by deriving Green-Kubo relations using time correlation functions of the generalized flux variables [2, 11]. The procedure is analogous, with the resulting transport equations derived in Epstein and Mandadapu

$$\begin{aligned}\rho\dot{\mathbf{v}} =& \lambda_1 \nabla(\nabla\cdot\mathbf{v}) + \lambda_2 \boldsymbol{\Delta}\mathbf{v} + (\lambda_5 - \lambda_6)\,\boldsymbol{\epsilon}\cdot\nabla(\nabla\cdot\mathbf{v}) \\ &+ (\lambda_4 + \lambda_5 + \lambda_6)\,\boldsymbol{\epsilon}\cdot\boldsymbol{\Delta}\mathbf{v} + \boldsymbol{\epsilon}\cdot\nabla(\gamma_2 m - \lambda_3 \nabla\times\mathbf{v}) \\ &+ \nabla\left[\gamma_1 m - (\lambda_5 - \lambda_6)\nabla\times\mathbf{v}\right].\end{aligned} \tag{15}$$

These reduce to the ones used in the manuscript, e.g. the 2D case with no internal spins $m = \lambda_3 = \lambda_5 = 0$:

$$\begin{aligned}&\lambda_1 \to \frac{1}{2}(\alpha_{1111} + \alpha_{1122}), \quad \lambda_2 \to \frac{1}{2}(\alpha_{1111} - \alpha_{1122}), \\ &\lambda_4 \to \beta_{1112}, \quad \lambda_6 \to \frac{1}{2}\gamma_{1112}.\end{aligned} \tag{16}$$

Momentum Relaxing Body Force. Strictly speaking, Cauchy's laws of motion, given by eqs. (1) and (2), arise as a consequence of momentum conservation. Electrons in condensed matter however, especially at elevated temperatures, can undergo multiple momentum-relaxing scattering events, e.g. against impurities or phonons. We incorporate such momentum-relaxation in the manuscript by adding a body force term of the form:

$$\rho u_j \partial_j u_i = -\partial_i p + \partial_j \tau_{ji} - R_{ij} u_j, \tag{17}$$

where $\mathbf{R}$ is a rank-two, positive-semidefinite tensor which is inversely proportional to a microscopic momentum-relaxing lifetime. In order to highlight the viscous effects of crystal anisotropy, the steady-state solutions shown in the manuscript (with the exception of Fig.1(e) justifying this choice) assumed $\mathbf{R} \to 0$. In this section, we investigate the effects of this term.

First, we establish a baseline effect for the isotropic (SO(2)) case in our annulus geometry, where the momentum relaxing term is given by $R_{ij} = \delta_{ij}$. Since both the viscous and momentum-relaxing terms are isotropic, the velocity profile is angularly symmetric (Supplementary Fig. 1(a)). As the relative magnitude of the momentum relaxing term increases, the flow dissipates quicker, resulting in a significant part of the annulus exhibiting stationary flow for $|\mathbf{R}|/|\mathbf{A}| > 1$. Next, we investigate the case where the viscous and momentum-relaxing terms have different symmetries. According to Neumann's principle, this is allowed by the difference in rank in the two material property tensors. For example, in the square (D_4) point group, while the viscous tensor exhibits anisotropy, the momentum relaxing term is still given by $R_{ij} = \delta_{ij}$. As such, the underlying four-fold symmetry of the steady-state vortices remains the same (Supplementary Fig.1(b-e)), with the momentum-relaxing term smearing out these vortices for $|R|/|A| > 1$ (Supplementary Fig.1(f)). The magnitude of the vortices however, is largely unchanged at $\sim 12.5\%$, indicating the effect to be as observable.

Finally, we investigate the effects of momentum-relaxation on a purely hydrodynamic phenomenon we proposed, namely the pressure gauge. Supplementary Figure 2 shows that the effect of momentum-relaxation on the pressure drop used to quantify objectivity-breaking terms in the manuscript. While for values of $|R|/|A| < 0.1$ the momentum-relaxing term doesn't alter the results significantly, the pressure drop is no longer directly proportional to the objectivity-breaking terms for $|R|/|A| > 0.1$.

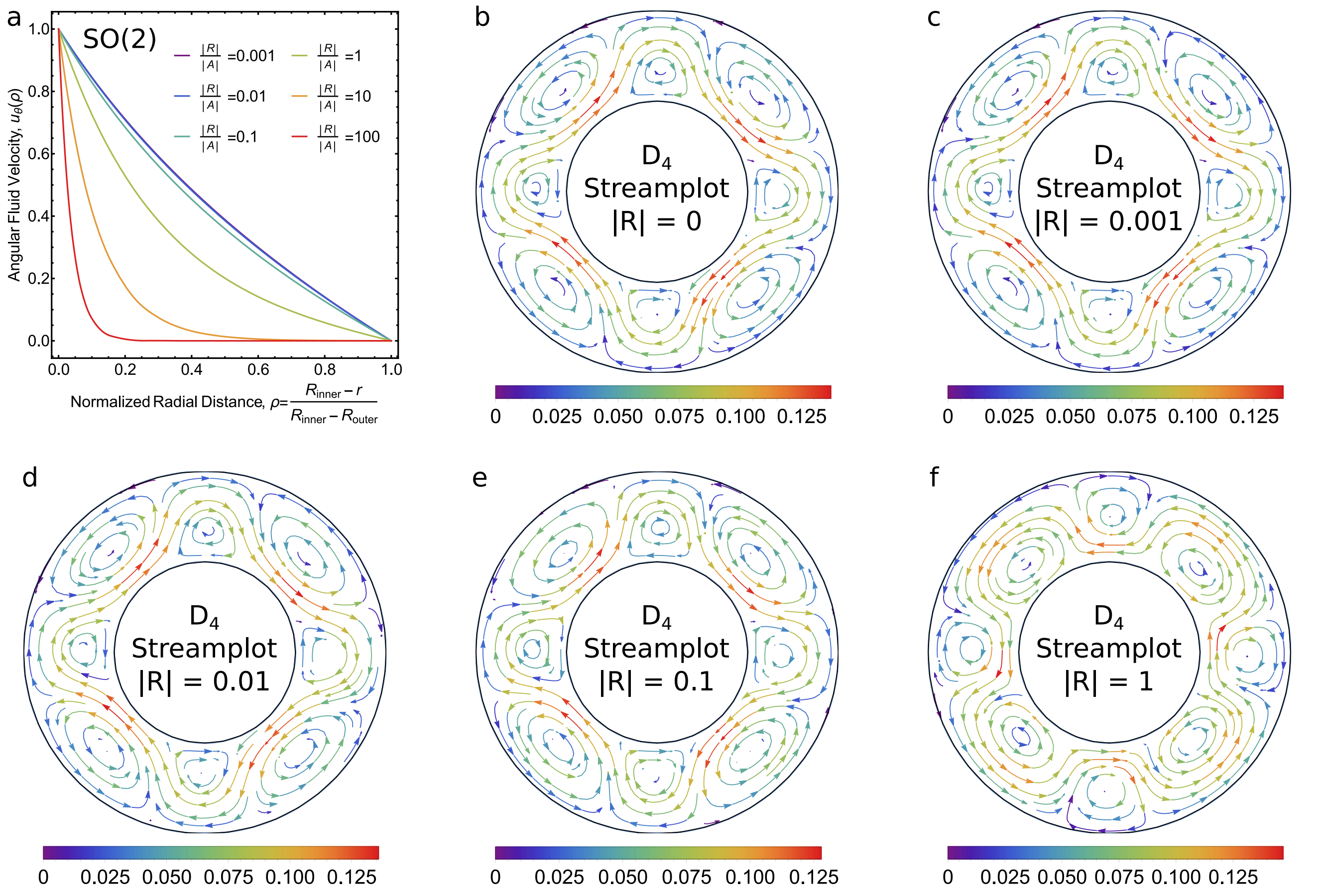

Fig. 1. **Magnitude Effects of Momentum Relaxing Terms on Steady State Vortices a** Steady state angular fluid velocity for various normalized values of the momentum relaxing term in an isotropic annulus. The flow becomes nearly-stationary in much of the annulus for $|R|/|A| > 1$. **(b-f)** Steady state streamplots difference between isotropic flows and flows in square (D_4) systems for various normalized values of the momentum relaxing term. The effects are hardly noticable for values of $|R|/|A| < 1$, with the steady-state vortices getting smeared out, albeit still observable, for $|R|/|A| = 1$. Color-scales indicate magnitude of the velocity vector field.

Neumann's Principle. In this section, we demonstrate the application of Neumann's principle on the rank-4 viscosity tensor. For convenience, we restrict ourselves in a two-dimensional projection (xy-plane) of the most general symbolic viscosity tensor in three dimensions, with ij-symmetry, given by:

$$A^{(001)} = \begin{pmatrix} \begin{pmatrix} \alpha_{1111} & \alpha_{1112}+\beta_{1112}+\gamma_{1112} \\ \alpha_{1112}+\beta_{1112}-\gamma_{1112} & \alpha_{1122}+\beta_{1122} \end{pmatrix} & \begin{pmatrix} \alpha_{1112}-\beta_{1112} & \alpha_{1212}+\gamma_{1212} \\ \alpha_{1212}-\gamma_{1212} & \alpha_{1222}+\beta_{1222} \end{pmatrix} \\ \begin{pmatrix} \alpha_{1112}-\beta_{1112} & \alpha_{1212}+\gamma_{1212} \\ \alpha_{1212}-\gamma_{1212} & \alpha_{1222}+\beta_{1222} \end{pmatrix} & \begin{pmatrix} \alpha_{1122}-\beta_{1122} & \alpha_{1222}-\beta_{1222}+\gamma_{2212} \\ \alpha_{1222}-\beta_{1222}-\gamma_{2212} & \alpha_{2222} \end{pmatrix} \end{pmatrix}, \tag{18}$$

and take into account the effects of four-fold (C_4) and square (D_4) symmetry elements given below:

C_4				D_4							
E	$\underline{C_4}$	C_2	C_4^3	E	$\underline{C_4}$	C_2	C_4^3	$\underline{C_2^x}$	C_2^y	C_2^{x+y}	C_2^{x-y}

In general, we only need to consider the minimal set of symmetry generators, underlined for each point group above.

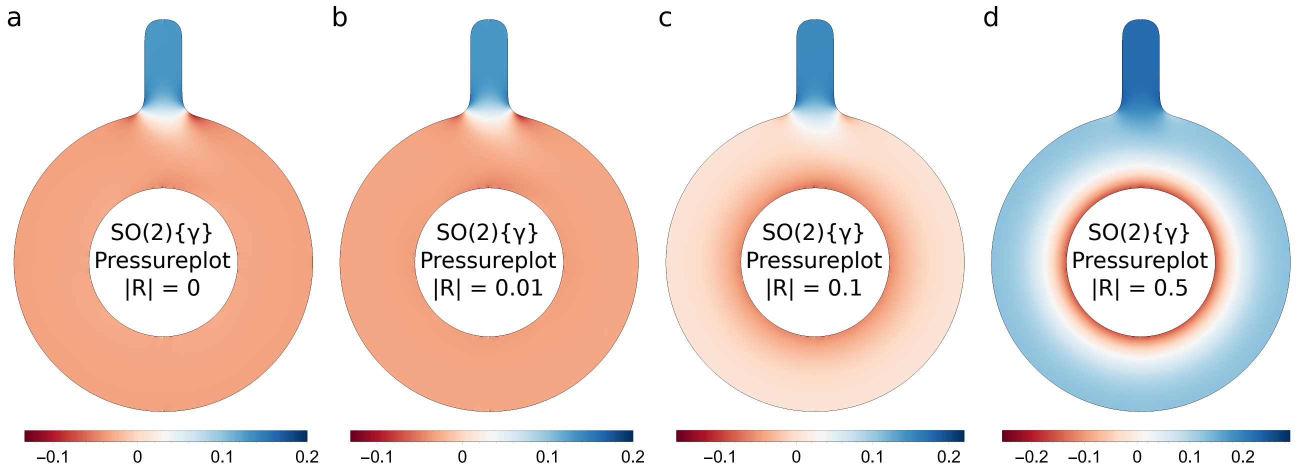

Fig. 2. **Magnitude Effects of Momentum Relaxing Terms on Pressure Gauge** Steady state pressure difference between the viscosity tensor and the same with additional stress objectivity breaking terms for various normalized values of the momentum relaxing term. The effects are noticable for values of $|R|/|A| > 0.1$. Color-scales indicate magnitude of the pressure field.

Applying the C_4 operation to eq. (18) according to the transformation law, we obtain:

$$A'^{(001)} = \begin{pmatrix} \begin{pmatrix} \alpha_{2222} & -\alpha_{1222}+\beta_{1222}+\gamma_{2212} \\ -\alpha_{1222}+\beta_{1222}-\gamma_{2212} & \alpha_{1122}-\beta_{1122} \end{pmatrix} & \begin{pmatrix} -\alpha_{1222}-\beta_{1222} & \alpha_{1212}-\gamma_{1212} \\ \alpha_{1212}+\gamma_{1212} & \beta_{1112}-\alpha_{1112} \end{pmatrix} \\ \begin{pmatrix} -\alpha_{1222}-\beta_{1222} & \alpha_{1212}-\gamma_{1212} \\ \alpha_{1212}+\gamma_{1212} & \beta_{1112}-\alpha_{1112} \end{pmatrix} & \begin{pmatrix} \alpha_{1122}+\beta_{1122} & -\alpha_{1112}-\beta_{1112}+\gamma_{1112} \\ -\alpha_{1112}-\beta_{1112}-\gamma_{1112} & \alpha_{1111} \end{pmatrix} \end{pmatrix}. \quad (19)$$

Setting eqs. (18) and (19) equal to each other, reduces the number of independent coefficients from 12 to 6

$$A^{C_4^{(001)}} = \begin{pmatrix} \begin{pmatrix} \alpha_{1111} & \alpha_{1112}+\beta_{1112}+\gamma_{1112} \\ \alpha_{1112}+\beta_{1112}-\gamma_{1112} & \alpha_{1122} \end{pmatrix} & \begin{pmatrix} \alpha_{1112}-\beta_{1112} & \alpha_{1212} \\ \alpha_{1212} & \beta_{1112}-\alpha_{1112} \end{pmatrix} \\ \begin{pmatrix} \alpha_{1112}-\beta_{1112} & \alpha_{1212} \\ \alpha_{1212} & \beta_{1112}-\alpha_{1112} \end{pmatrix} & \begin{pmatrix} \alpha_{1122} & -\alpha_{1112}-\beta_{1112}+\gamma_{1112} \\ -\alpha_{1112}-\beta_{1112}-\gamma_{1112} & \alpha_{1111} \end{pmatrix} \end{pmatrix}. \quad (20)$$

Similarly, acting on eq. (18) with both the C_4 and C_2^x symmetry operators, further reduces the number of coefficients to 3

$$A^{D_4^{(001)}} = \begin{pmatrix} \begin{pmatrix} \alpha_{1111} & 0 \\ 0 & \alpha_{1122} \end{pmatrix} & \begin{pmatrix} 0 & \alpha_{1212} \\ \alpha_{1212} & 0 \end{pmatrix} \\ \begin{pmatrix} 0 & \alpha_{1212} \\ \alpha_{1212} & 0 \end{pmatrix} & \begin{pmatrix} \alpha_{1122} & 0 \\ 0 & \alpha_{1111} \end{pmatrix} \end{pmatrix}. \quad (21)$$

The remaining three coefficients can be recovered using the following parametrization:

$$A_{ijkl}^{C_4^{(001)}} = A_{ijkl}^{D_4^{(001)}} + \mathcal{A}\left(\sigma_{ij}^x\sigma_{kl}^z + \sigma_{ij}^z\sigma_{kl}^x\right) + \mathcal{B}\left(\delta_{ik}\epsilon_{jl} + \delta_{jl}\epsilon_{ik}\right) + \Gamma\delta_{ij}\epsilon_{kl}. \quad (22)$$

Numerical Details. All steady-state solutions of the Navier-Stokes equation were obtained using the nonlinear finite element solver of the computational package Wolfram Mathematica 12.0 [12]. The mesh resolution was 10978, 46290, and 25052 triangle elements for the annulus, pressure-gauge, and expanding channel geometries respectively. The viscosity tensors used

were as follows:

$$A^{\mathrm{SO}(2)} = \begin{pmatrix} \begin{pmatrix} 0.0745043 & 0.587221 \\ -0.0483742 & 0.0118486 \end{pmatrix} & \begin{pmatrix} -0.269423 & 0.0313279 \\ 0.0313279 & 0.269423 \end{pmatrix} \\ \begin{pmatrix} -0.269423 & 0.0313279 \\ 0.0313279 & 0.269423 \end{pmatrix} & \begin{pmatrix} 0.0118486 & 0.0483742 \\ -0.587221 & 0.0745043 \end{pmatrix} \end{pmatrix},$$

$$A^{D_6} = \begin{pmatrix} \begin{pmatrix} 0.289547 & 0.398427 \\ 0.189569 & 0.213894 \end{pmatrix} & \begin{pmatrix} -0.293998 & 0.0378261 \\ 0.0378261 & 0.293998 \end{pmatrix} \\ \begin{pmatrix} -0.293998 & 0.0378261 \\ 0.0378261 & 0.293998 \end{pmatrix} & \begin{pmatrix} 0.213894 & -0.189569 \\ -0.398427 & 0.289547 \end{pmatrix} \end{pmatrix},$$

$$A^{D_4} = \begin{pmatrix} \begin{pmatrix} 0.160842 & 0.369235 \\ -0.106286 & 0.0885322 \end{pmatrix} & \begin{pmatrix} -0.131474 & 0.376888 \\ 0.376888 & 0.131474 \end{pmatrix} \\ \begin{pmatrix} -0.131474 & 0.376888 \\ 0.376888 & 0.131474 \end{pmatrix} & \begin{pmatrix} 0.0885322 & 0.106286 \\ -0.369235 & 0.160842 \end{pmatrix} \end{pmatrix},$$

$$A^{D_{2h}^{(001)}} = \begin{pmatrix} \begin{pmatrix} 0.302739 & 0 \\ 0 & 0.119665 \end{pmatrix} & \begin{pmatrix} 0 & 0.404212 \\ 0.404212 & 0 \end{pmatrix} \\ \begin{pmatrix} 0 & 0.404212 \\ 0.404212 & 0 \end{pmatrix} & \begin{pmatrix} 0.119665 & 0 \\ 0 & 0.475563 \end{pmatrix} \end{pmatrix},$$

$$A^{O_h^{(111)}} = \begin{pmatrix} \begin{pmatrix} 0.651174 & 0. \\ 0. & 0.000836994 \end{pmatrix} & \begin{pmatrix} 0. & 0.325169 \\ 0.325169 & 0. \end{pmatrix} \\ \begin{pmatrix} 0. & 0.325169 \\ 0.325169 & 0. \end{pmatrix} & \begin{pmatrix} 0.000836994 & 0. \\ 0. & 0.651174 \end{pmatrix} \end{pmatrix},$$

$$A^{O_h^{(101)}} = \begin{pmatrix} \begin{pmatrix} 0.651174 & 0. \\ 0. & 0.0872965 \end{pmatrix} & \begin{pmatrix} 0. & 0.411628 \\ 0.411628 & 0. \end{pmatrix} \\ \begin{pmatrix} 0. & 0.411628 \\ 0.411628 & 0. \end{pmatrix} & \begin{pmatrix} 0.0872965 & 0. \\ 0. & 0.391796 \end{pmatrix} \end{pmatrix},$$

$$A^{O_h^{(001)}} = \begin{pmatrix} \begin{pmatrix} 0.391796 & 0. \\ 0. & 0.0872965 \end{pmatrix} & \begin{pmatrix} 0. & 0.411628 \\ 0.411628 & 0. \end{pmatrix} \\ \begin{pmatrix} 0. & 0.411628 \\ 0.411628 & 0. \end{pmatrix} & \begin{pmatrix} 0.0872965 & 0. \\ 0. & 0.391796 \end{pmatrix} \end{pmatrix},$$

Supplementary Notes

Symbolic 2D Viscosity Tensors. In two dimensions, the 10 point groups reduce to 5 different viscosity tensor classes:

C_1 (Oblique) & C_2 (Rectangular)

$$\begin{pmatrix} \begin{pmatrix} \alpha_{1111} & \alpha_{1112}+\beta_{1112}+\gamma_{1112} \\ \alpha_{1112}+\beta_{1112}-\gamma_{1112} & \alpha_{1122}+\beta_{1122} \end{pmatrix} & \begin{pmatrix} \alpha_{1112}-\beta_{1112} & \alpha_{1212}+\gamma_{1212} \\ \alpha_{1212}-\gamma_{1212} & \alpha_{1222}+\beta_{1222} \end{pmatrix} \\ \begin{pmatrix} \alpha_{1112}-\beta_{1112} & \alpha_{1212}+\gamma_{1212} \\ \alpha_{1212}-\gamma_{1212} & \alpha_{1222}+\beta_{1222} \end{pmatrix} & \begin{pmatrix} \alpha_{1122}-\beta_{1122} & \alpha_{1222}-\beta_{1222}+\gamma_{2212} \\ \alpha_{1222}-\beta_{1222}-\gamma_{2212} & \alpha_{2222} \end{pmatrix} \end{pmatrix}$$

D_1(Oblique) & D_2 (Rectangular)

$$\begin{pmatrix} \begin{pmatrix} \alpha_{1111} & \beta_{1112}+\gamma_{1112} \\ \beta_{1112}-\gamma_{1112} & \alpha_{1122} \end{pmatrix} & \begin{pmatrix} -\beta_{1112} & \alpha_{1212} \\ \alpha_{1212} & \beta_{1222} \end{pmatrix} \\ \begin{pmatrix} -\beta_{1112} & \alpha_{1212} \\ \alpha_{1212} & \beta_{1222} \end{pmatrix} & \begin{pmatrix} \alpha_{1122} & \gamma_{2212}-\beta_{1222} \\ -\beta_{1222}-\gamma_{2212} & \alpha_{2222} \end{pmatrix} \end{pmatrix}$$

C_4 (Square)

$$\begin{pmatrix} \begin{pmatrix} \alpha_{1111} & \alpha_{1112}+\beta_{1112}+\gamma_{1112} \\ \alpha_{1112}+\beta_{1112}-\gamma_{1112} & \alpha_{1122} \end{pmatrix} & \begin{pmatrix} \alpha_{1112}-\beta_{1112} & \alpha_{1212} \\ \alpha_{1212} & \beta_{1112}-\alpha_{1112} \end{pmatrix} \\ \begin{pmatrix} \alpha_{1112}-\beta_{1112} & \alpha_{1212} \\ \alpha_{1212} & \beta_{1112}-\alpha_{1112} \end{pmatrix} & \begin{pmatrix} \alpha_{1122} & -\alpha_{1112}-\beta_{1112}+\gamma_{1112} \\ -\alpha_{1112}-\beta_{1112}-\gamma_{1112} & \alpha_{1111} \end{pmatrix} \end{pmatrix}$$

D_4 (Square)

$$\begin{pmatrix} \begin{pmatrix} \alpha_{1111} & \beta_{1112}+\gamma_{1112} \\ \beta_{1112}-\gamma_{1112} & \alpha_{1122} \end{pmatrix} & \begin{pmatrix} -\beta_{1112} & \alpha_{1212} \\ \alpha_{1212} & \beta_{1112} \end{pmatrix} \\ \begin{pmatrix} -\beta_{1112} & \alpha_{1212} \\ \alpha_{1212} & \beta_{1112} \end{pmatrix} & \begin{pmatrix} \alpha_{1122} & \gamma_{1112}-\beta_{1112} \\ -\beta_{1112}-\gamma_{1112} & \alpha_{1111} \end{pmatrix} \end{pmatrix}$$

C_3,C_6,D_3,D_6 (Hexagonal) & $SO(2)$ (Isotropic)

$$\begin{pmatrix} \begin{pmatrix} \alpha_{1111} & \beta_{1112}+\gamma_{1112} \\ \beta_{1112}-\gamma_{1112} & \alpha_{1122} \end{pmatrix} & \begin{pmatrix} -\beta_{1112} & \frac{1}{2}(\alpha_{1111}-\alpha_{1122}) \\ \frac{1}{2}(\alpha_{1111}-\alpha_{1122}) & \beta_{1112} \end{pmatrix} \\ \begin{pmatrix} -\beta_{1112} & \frac{1}{2}(\alpha_{1111}-\alpha_{1122}) \\ \frac{1}{2}(\alpha_{1111}-\alpha_{1122}) & \beta_{1112} \end{pmatrix} & \begin{pmatrix} \alpha_{1122} & \gamma_{1112}-\beta_{1112} \\ -\beta_{1112}-\gamma_{1112} & \alpha_{1111} \end{pmatrix} \end{pmatrix}$$

Symbolic 3D Viscosity Tensors. In three dimensions, the 32 point groups reduce to 28 different viscosity tensor classes, with $C_{6\mathrm{h}} = D_{6\mathrm{h}}$, $T = T_\mathrm{d}$ and $T_\mathrm{h} = O = O_\mathrm{h}$ having identical tensors. We present only the hexagonal, cubic, and isotropic classes here.

C_6 (Hexagonal)

$$\begin{pmatrix} \begin{pmatrix} \alpha_{1111} & \beta_{1112}+\gamma_{1112} & 0 \\ \beta_{1112}-\gamma_{1112} & \alpha_{1122} & 0 \\ 0 & 0 & \alpha_{1133}+\beta_{1133} \end{pmatrix} & \begin{pmatrix} -\beta_{1112} & \frac{1}{2}(\alpha_{1111}-\alpha_{1122}) & 0 \\ \frac{1}{2}(\alpha_{1111}-\alpha_{1122}) & \beta_{1112} & 0 \\ 0 & 0 & 0 \end{pmatrix} & \begin{pmatrix} 0 & 0 & \alpha_{1313}+\gamma_{1313} \\ 0 & 0 & \beta_{1323}+\gamma_{1323} \\ \alpha_{1313}-\gamma_{1313} & \beta_{1323}-\gamma_{1323} & 0 \end{pmatrix} \\ \begin{pmatrix} -\beta_{1112} & \frac{1}{2}(\alpha_{1111}-\alpha_{1122}) & 0 \\ \frac{1}{2}(\alpha_{1111}-\alpha_{1122}) & \beta_{1112} & 0 \\ 0 & 0 & 0 \end{pmatrix} & \begin{pmatrix} \alpha_{1122} & \gamma_{1112}-\beta_{1112} & 0 \\ -\beta_{1112}-\gamma_{1112} & \alpha_{1111} & 0 \\ 0 & 0 & \alpha_{1133}+\beta_{1133} \end{pmatrix} & \begin{pmatrix} 0 & 0 & -\beta_{1323}-\gamma_{1323} \\ 0 & 0 & \alpha_{1313}+\gamma_{1313} \\ \gamma_{1323}-\beta_{1323} & \alpha_{1313}-\gamma_{1313} & 0 \end{pmatrix} \\ \begin{pmatrix} 0 & 0 & \alpha_{1313}+\gamma_{1313} \\ 0 & 0 & \beta_{1323}+\gamma_{1323} \\ \alpha_{1313}-\gamma_{1313} & \beta_{1323}-\gamma_{1323} & 0 \end{pmatrix} & \begin{pmatrix} 0 & 0 & -\beta_{1323}-\gamma_{1323} \\ 0 & 0 & \alpha_{1313}+\gamma_{1313} \\ \gamma_{1323}-\beta_{1323} & \alpha_{1313}-\gamma_{1313} & 0 \end{pmatrix} & \begin{pmatrix} \alpha_{1133}-\beta_{1133} & \gamma_{3312} & 0 \\ -\gamma_{3312} & \alpha_{1133}-\beta_{1133} & 0 \\ 0 & 0 & \alpha_{3333} \end{pmatrix} \end{pmatrix}$$

C_{6v} (Hexagonal)

$$\begin{pmatrix} \begin{pmatrix} \alpha_{1111} & \beta_{1112}+\gamma_{1112} & 0 \\ \beta_{1112}-\gamma_{1112} & \alpha_{1122} & 0 \\ 0 & 0 & \alpha_{1133} \end{pmatrix} & \begin{pmatrix} -\beta_{1112} & \frac{1}{2}(\alpha_{1111}-\alpha_{1122}) & 0 \\ \frac{1}{2}(\alpha_{1111}-\alpha_{1122}) & \beta_{1112} & 0 \\ 0 & 0 & 0 \end{pmatrix} & \begin{pmatrix} 0 & 0 & \alpha_{1313} \\ 0 & 0 & \beta_{1323}+\gamma_{1323} \\ \alpha_{1313} & \beta_{1323}-\gamma_{1323} & 0 \end{pmatrix} \\ \begin{pmatrix} -\beta_{1112} & \frac{1}{2}(\alpha_{1111}-\alpha_{1122}) & 0 \\ \frac{1}{2}(\alpha_{1111}-\alpha_{1122}) & \beta_{1112} & 0 \\ 0 & 0 & 0 \end{pmatrix} & \begin{pmatrix} \alpha_{1122} & \gamma_{1112}-\beta_{1112} & 0 \\ -\beta_{1112}-\gamma_{1112} & \alpha_{1111} & 0 \\ 0 & 0 & \alpha_{1133} \end{pmatrix} & \begin{pmatrix} 0 & 0 & -\beta_{1323}-\gamma_{1323} \\ 0 & 0 & \alpha_{1313} \\ \gamma_{1323}-\beta_{1323} & \alpha_{1313} & 0 \end{pmatrix} \\ \begin{pmatrix} 0 & 0 & \alpha_{1313} \\ 0 & 0 & \beta_{1323}+\gamma_{1323} \\ \alpha_{1313} & \beta_{1323}-\gamma_{1323} & 0 \end{pmatrix} & \begin{pmatrix} 0 & 0 & -\beta_{1323}-\gamma_{1323} \\ 0 & 0 & \alpha_{1313} \\ \gamma_{1323}-\beta_{1323} & \alpha_{1313} & 0 \end{pmatrix} & \begin{pmatrix} \alpha_{1133} & \gamma_{3312} & 0 \\ -\gamma_{3312} & \alpha_{1133} & 0 \\ 0 & 0 & \alpha_{3333} \end{pmatrix} \end{pmatrix}$$

C_{3h} (Hexagonal)

$$\begin{pmatrix} \begin{pmatrix} \alpha_{1111} & 0 & \alpha_{1113} \\ 0 & \alpha_{1122} & \alpha_{1123} \\ \alpha_{1113} & \alpha_{1123} & \alpha_{1133} \end{pmatrix} & \begin{pmatrix} 0 & \frac{1}{2}(\alpha_{1111}-\alpha_{1122}) & \alpha_{1123} \\ \frac{1}{2}(\alpha_{1111}-\alpha_{1122}) & 0 & -\alpha_{1113} \\ \alpha_{1123} & -\alpha_{1113} & 0 \end{pmatrix} & \begin{pmatrix} \alpha_{1113} & \alpha_{1123} & \alpha_{1313} \\ \alpha_{1123} & -\alpha_{1113} & 0 \\ \alpha_{1313} & 0 & 0 \end{pmatrix} \\ \begin{pmatrix} 0 & \frac{1}{2}(\alpha_{1111}-\alpha_{1122}) & \alpha_{1123} \\ \frac{1}{2}(\alpha_{1111}-\alpha_{1122}) & 0 & -\alpha_{1113} \\ \alpha_{1123} & -\alpha_{1113} & 0 \end{pmatrix} & \begin{pmatrix} \alpha_{1122} & 0 & -\alpha_{1113} \\ 0 & \alpha_{1111} & -\alpha_{1123} \\ -\alpha_{1113} & -\alpha_{1123} & \alpha_{1133} \end{pmatrix} & \begin{pmatrix} \alpha_{1123} & -\alpha_{1113} & 0 \\ -\alpha_{1113} & -\alpha_{1123} & \alpha_{1313} \\ 0 & \alpha_{1313} & 0 \end{pmatrix} \\ \begin{pmatrix} \alpha_{1113} & \alpha_{1123} & \alpha_{1313} \\ \alpha_{1123} & -\alpha_{1113} & 0 \\ \alpha_{1313} & 0 & 0 \end{pmatrix} & \begin{pmatrix} \alpha_{1123} & -\alpha_{1113} & 0 \\ -\alpha_{1113} & -\alpha_{1123} & \alpha_{1313} \\ 0 & \alpha_{1313} & 0 \end{pmatrix} & \begin{pmatrix} \alpha_{1133} & 0 & 0 \\ 0 & \alpha_{1133} & 0 \\ 0 & 0 & \alpha_{3333} \end{pmatrix} \end{pmatrix}$$

D_{3h} (Hexagonal)

$$
\begin{pmatrix}
\begin{pmatrix} \alpha_{1111} & 0 & \alpha_{1213} \\ 0 & \alpha_{1122} & \alpha_{1213} \\ \alpha_{1213} & \alpha_{1213} & \alpha_{1133} \end{pmatrix} &
\begin{pmatrix} 0 & \frac{1}{2}\left(\alpha_{1111}-\alpha_{1122}\right) & \alpha_{1213} \\ \frac{1}{2}\left(\alpha_{1111}-\alpha_{1122}\right) & 0 & -\alpha_{1213} \\ \alpha_{1213} & -\alpha_{1213} & 0 \end{pmatrix} &
\begin{pmatrix} \alpha_{1213} & \alpha_{1213} & \alpha_{1313} \\ \alpha_{1213} & -\alpha_{1213} & 0 \\ \alpha_{1313} & 0 & 0 \end{pmatrix} \\
\begin{pmatrix} 0 & \frac{1}{2}\left(\alpha_{1111}-\alpha_{1122}\right) & \alpha_{1213} \\ \frac{1}{2}\left(\alpha_{1111}-\alpha_{1122}\right) & 0 & -\alpha_{1213} \\ \alpha_{1213} & -\alpha_{1213} & 0 \end{pmatrix} &
\begin{pmatrix} \alpha_{1122} & 0 & -\alpha_{1213} \\ 0 & \alpha_{1111} & -\alpha_{1213} \\ -\alpha_{1213} & -\alpha_{1213} & \alpha_{1133} \end{pmatrix} &
\begin{pmatrix} \alpha_{1213} & -\alpha_{1213} & 0 \\ -\alpha_{1213} & -\alpha_{1213} & \alpha_{1313} \\ 0 & \alpha_{1313} & 0 \end{pmatrix} \\
\begin{pmatrix} \alpha_{1213} & \alpha_{1213} & \alpha_{1313} \\ \alpha_{1213} & -\alpha_{1213} & 0 \\ \alpha_{1313} & 0 & 0 \end{pmatrix} &
\begin{pmatrix} \alpha_{1213} & -\alpha_{1213} & 0 \\ -\alpha_{1213} & -\alpha_{1213} & \alpha_{1313} \\ 0 & \alpha_{1313} & 0 \end{pmatrix} &
\begin{pmatrix} \alpha_{1133} & 0 & 0 \\ 0 & \alpha_{1133} & 0 \\ 0 & 0 & \alpha_{3333} \end{pmatrix}
\end{pmatrix}
$$

D_6 (Hexagonal)

$$
\begin{pmatrix}
\begin{pmatrix} \alpha_{1111} & 0 & 0 \\ 0 & \alpha_{1122} & 0 \\ 0 & 0 & \alpha_{1133}+\beta_{1133} \end{pmatrix} &
\begin{pmatrix} 0 & \frac{1}{2}\left(\alpha_{1111}-\alpha_{1122}\right) & 0 \\ \frac{1}{2}\left(\alpha_{1111}-\alpha_{1122}\right) & 0 & 0 \\ 0 & 0 & 0 \end{pmatrix} &
\begin{pmatrix} 0 & 0 & \alpha_{1313}+\gamma_{1313} \\ 0 & 0 & 0 \\ \alpha_{1313}-\gamma_{1313} & 0 & 0 \end{pmatrix} \\
\begin{pmatrix} 0 & \frac{1}{2}\left(\alpha_{1111}-\alpha_{1122}\right) & 0 \\ \frac{1}{2}\left(\alpha_{1111}-\alpha_{1122}\right) & 0 & 0 \\ 0 & 0 & 0 \end{pmatrix} &
\begin{pmatrix} \alpha_{1122} & 0 & 0 \\ 0 & \alpha_{1111} & 0 \\ 0 & 0 & \alpha_{1133}+\beta_{1133} \end{pmatrix} &
\begin{pmatrix} 0 & 0 & 0 \\ 0 & 0 & \alpha_{1313}+\gamma_{1313} \\ 0 & \alpha_{1313}-\gamma_{1313} & 0 \end{pmatrix} \\
\begin{pmatrix} 0 & 0 & \alpha_{1313}+\gamma_{1313} \\ 0 & 0 & 0 \\ \alpha_{1313}-\gamma_{1313} & 0 & 0 \end{pmatrix} &
\begin{pmatrix} 0 & 0 & 0 \\ 0 & 0 & \alpha_{1313}+\gamma_{1313} \\ 0 & \alpha_{1313}-\gamma_{1313} & 0 \end{pmatrix} &
\begin{pmatrix} \alpha_{1133}-\beta_{1133} & 0 & 0 \\ 0 & \alpha_{1133}-\beta_{1133} & 0 \\ 0 & 0 & \alpha_{3333} \end{pmatrix}
\end{pmatrix}
$$

C_{6h} & D_{6h} (Hexagonal)

$$
\begin{pmatrix}
\begin{pmatrix} \alpha_{1111} & 0 & 0 \\ 0 & \alpha_{1122} & 0 \\ 0 & 0 & \alpha_{1133} \end{pmatrix} &
\begin{pmatrix} 0 & \frac{1}{2}\left(\alpha_{1111}-\alpha_{1122}\right) & 0 \\ \frac{1}{2}\left(\alpha_{1111}-\alpha_{1122}\right) & 0 & 0 \\ 0 & 0 & 0 \end{pmatrix} &
\begin{pmatrix} 0 & 0 & \alpha_{1313} \\ 0 & 0 & 0 \\ \alpha_{1313} & 0 & 0 \end{pmatrix} \\
\begin{pmatrix} 0 & \frac{1}{2}\left(\alpha_{1111}-\alpha_{1122}\right) & 0 \\ \frac{1}{2}\left(\alpha_{1111}-\alpha_{1122}\right) & 0 & 0 \\ 0 & 0 & 0 \end{pmatrix} &
\begin{pmatrix} \alpha_{1122} & 0 & 0 \\ 0 & \alpha_{1111} & 0 \\ 0 & 0 & \alpha_{1133} \end{pmatrix} &
\begin{pmatrix} 0 & 0 & 0 \\ 0 & 0 & \alpha_{1313} \\ 0 & \alpha_{1313} & 0 \end{pmatrix} \\
\begin{pmatrix} 0 & 0 & \alpha_{1313} \\ 0 & 0 & 0 \\ \alpha_{1313} & 0 & 0 \end{pmatrix} &
\begin{pmatrix} 0 & 0 & 0 \\ 0 & 0 & \alpha_{1313} \\ 0 & \alpha_{1313} & 0 \end{pmatrix} &
\begin{pmatrix} \alpha_{1133} & 0 & 0 \\ 0 & \alpha_{1133} & 0 \\ 0 & 0 & \alpha_{3333} \end{pmatrix}
\end{pmatrix}
$$

T &T_d (Cubic)

$$
\begin{pmatrix}
\begin{pmatrix} \alpha_{1111} & 0 & 0 \\ 0 & \alpha_{1122}+\beta_{1122} & 0 \\ 0 & 0 & \alpha_{1122}-\beta_{1122} \end{pmatrix} &
\begin{pmatrix} 0 & \alpha_{1212}+\gamma_{1212} & 0 \\ \alpha_{1212}-\gamma_{1212} & 0 & 0 \\ 0 & 0 & 0 \end{pmatrix} &
\begin{pmatrix} 0 & 0 & \alpha_{1212}-\gamma_{1212} \\ 0 & 0 & 0 \\ \alpha_{1212}+\gamma_{1212} & 0 & 0 \end{pmatrix} \\
\begin{pmatrix} 0 & \alpha_{1212}+\gamma_{1212} & 0 \\ \alpha_{1212}-\gamma_{1212} & 0 & 0 \\ 0 & 0 & 0 \end{pmatrix} &
\begin{pmatrix} \alpha_{1122}-\beta_{1122} & 0 & 0 \\ 0 & \alpha_{1111} & 0 \\ 0 & 0 & \alpha_{1122}+\beta_{1122} \end{pmatrix} &
\begin{pmatrix} 0 & 0 & 0 \\ 0 & 0 & \alpha_{1212}+\gamma_{1212} \\ 0 & \alpha_{1212}-\gamma_{1212} & 0 \end{pmatrix} \\
\begin{pmatrix} 0 & 0 & \alpha_{1212}-\gamma_{1212} \\ 0 & 0 & 0 \\ \alpha_{1212}+\gamma_{1212} & 0 & 0 \end{pmatrix} &
\begin{pmatrix} 0 & 0 & 0 \\ 0 & 0 & \alpha_{1212}+\gamma_{1212} \\ 0 & \alpha_{1212}-\gamma_{1212} & 0 \end{pmatrix} &
\begin{pmatrix} \alpha_{1122}+\beta_{1122} & 0 & 0 \\ 0 & \alpha_{1122}-\beta_{1122} & 0 \\ 0 & 0 & \alpha_{1111} \end{pmatrix}
\end{pmatrix}
$$

T_h, O & O_h (Cubic)

$$
\begin{pmatrix}
\begin{pmatrix} \alpha_{1111} & 0 & 0 \\ 0 & \alpha_{1122} & 0 \\ 0 & 0 & \alpha_{1122} \end{pmatrix} &
\begin{pmatrix} 0 & \alpha_{1212} & 0 \\ \alpha_{1212} & 0 & 0 \\ 0 & 0 & 0 \end{pmatrix} &
\begin{pmatrix} 0 & 0 & \alpha_{1212} \\ 0 & 0 & 0 \\ \alpha_{1212} & 0 & 0 \end{pmatrix} \\
\begin{pmatrix} 0 & \alpha_{1212} & 0 \\ \alpha_{1212} & 0 & 0 \\ 0 & 0 & 0 \end{pmatrix} &
\begin{pmatrix} \alpha_{1122} & 0 & 0 \\ 0 & \alpha_{1111} & 0 \\ 0 & 0 & \alpha_{1122} \end{pmatrix} &
\begin{pmatrix} 0 & 0 & 0 \\ 0 & 0 & \alpha_{1212} \\ 0 & \alpha_{1212} & 0 \end{pmatrix} \\
\begin{pmatrix} 0 & 0 & \alpha_{1212} \\ 0 & 0 & 0 \\ \alpha_{1212} & 0 & 0 \end{pmatrix} &
\begin{pmatrix} 0 & 0 & 0 \\ 0 & 0 & \alpha_{1212} \\ 0 & \alpha_{1212} & 0 \end{pmatrix} &
\begin{pmatrix} \alpha_{1122} & 0 & 0 \\ 0 & \alpha_{1122} & 0 \\ 0 & 0 & \alpha_{1111} \end{pmatrix}
\end{pmatrix}
$$

$SO(3)$ **(Isotropic)**

$$
\begin{pmatrix}
\begin{pmatrix} \alpha_{1111} & 0 & 0 \\ 0 & \alpha_{1122} & 0 \\ 0 & 0 & \alpha_{1122} \end{pmatrix} &
\begin{pmatrix} 0 & \frac{1}{2}(\alpha_{1111}-\alpha_{1122}) & 0 \\ \frac{1}{2}(\alpha_{1111}-\alpha_{1122}) & 0 & 0 \\ 0 & 0 & 0 \end{pmatrix} &
\begin{pmatrix} 0 & 0 & \frac{1}{2}(\alpha_{1111}-\alpha_{1122}) \\ 0 & 0 & 0 \\ \frac{1}{2}(\alpha_{1111}-\alpha_{1122}) & 0 & 0 \end{pmatrix} \\
\begin{pmatrix} 0 & \frac{1}{2}(\alpha_{1111}-\alpha_{1122}) & 0 \\ \frac{1}{2}(\alpha_{1111}-\alpha_{1122}) & 0 & 0 \\ 0 & 0 & 0 \end{pmatrix} &
\begin{pmatrix} \alpha_{1122} & 0 & 0 \\ 0 & \alpha_{1111} & 0 \\ 0 & 0 & \alpha_{1122} \end{pmatrix} &
\begin{pmatrix} 0 & 0 & 0 \\ 0 & 0 & \frac{1}{2}(\alpha_{1111}-\alpha_{1122}) \\ 0 & \frac{1}{2}(\alpha_{1111}-\alpha_{1122}) & 0 \end{pmatrix} \\
\begin{pmatrix} 0 & 0 & \frac{1}{2}(\alpha_{1111}-\alpha_{1122}) \\ 0 & 0 & 0 \\ \frac{1}{2}(\alpha_{1111}-\alpha_{1122}) & 0 & 0 \end{pmatrix} &
\begin{pmatrix} 0 & 0 & 0 \\ 0 & 0 & \frac{1}{2}(\alpha_{1111}-\alpha_{1122}) \\ 0 & \frac{1}{2}(\alpha_{1111}-\alpha_{1122}) & 0 \end{pmatrix} &
\begin{pmatrix} \alpha_{1122} & 0 & 0 \\ 0 & \alpha_{1122} & 0 \\ 0 & 0 & \alpha_{1111} \end{pmatrix}
\end{pmatrix}
$$

Supplementary References

[1] V. K. Stokes, Physics of Fluids **9**, 1709 (1966).
[2] J. M. Epstein and K. K. Mandadapu, http://arxiv.org/abs/1907.10041v1.
[3] P. C. Martin, O. Parodi, and P. S. Pershan, Physical Review A **6**, 2401 (1972).
[4] L. D. Landau, *Theory of Elasticity 7* (Elsevier LTD, Oxford, 2004).
[5] B. Lautrup, *Physics of Continuous Matter* (Taylor & Francis Ltd, 2019).
[6] L. Onsager, Physical Review **37**, 405 (1931).
[7] L. Onsager, Physical Review **38**, 2265 (1931).
[8] R. W. Balluffi, S. M. Allen, and W. C. Carter, *Kinetics of Materials* (John Wiley & Sons, Inc., 2005).
[9] B. D. Coleman and C. Truesdell, The Journal of Chemical Physics **33**, 28 (1960).
[10] T. Heimburg, Physical Chemistry Chemical Physics **19**, 17331 (2017).
[11] P. Rao and B. Bradlyn, Phys. Rev. X **10**, 021005 (2020).
[12] W. R. Inc., "Mathematica, Version 12.0," (2019), champaign, IL, 2019.
