# Supplementary material for: Electron hydrodynamics in anisotropic materials
Source: Nat Commun. 2020 Sep 18;11:4710. doi: 10.1038/s41467-020-18553-y (PMC7501241; doi:10.1038/s41467-020-18553-y)
Supplement: Supplementary file 2 — Peer Review File [file 41467_2020_18553_MOESM2_ESM.pdf]

Editorial Note: This manuscript has been previously reviewed at another journal that is not operating a transparent peer review scheme. This document only contains reviewer comments and rebuttal letters for versions considered at *Nature Communications*. Mentions of prior referee reports have been redacted.

Reviewers' Comments:

Reviewer #1:
Remarks to the Author:
I am satisfied with the changes made to the manuscript by authors and with their replies to previous referees' comments. I recommend publishing the revised manuscript in Nature communications.

Reviewer #2:
Remarks to the Author:
The points that I've raised in the first report were addressed in the revised version satisfactory. The only exception is that I recommend that the authors discuss possible materials where the experiments can be done, accompanying it with a quantitative estimates. If this is done, even briefly, I think that the paper deserves to be published in Nature Communications.
